# The mediating role of psychological capital between work stress and compassion fatigue among clinical nurses: a cross-sectional study

**DOI:** 10.3389/fpubh.2026.1896133

**Published:** 2026-09-03

**Authors:** Chenxi Huang, Dengbin Liao, Yunting Luo, Yiran Xu, Haoyang Luo, Lingxiao He

**Affiliations:** 1 Nursing Department of West China Hospital, Sichuan University/West China School of Nursing, Sichuan University, Chengdu, China; 2 Trauma Center, West China Hospital, Sichuan University/West China School of Nursing, Sichuan University, Chengdu, China; 3 Center of Infectious Diseases, West China Hospital, Sichuan University/West China School of Nursing, Sichuan University, Chengdu, China; 4 Second People's Hospital of Jinniu District, Chengdu, China

**Keywords:** clinical nurses, compassion fatigue, psychological capital, resilience, work stress

## Abstract

**Objective:**

To explore the parallel multiple mediating effects of the sub-dimensions of psychological capital (self-efficacy, hope, resilience, and optimism) between work stress and compassion fatigue among clinical nurses.

**Methods:**

A cross-sectional study was conducted using convenience sampling. Clinical nurses from a tertiary hospital were recruited. Data were collected using a general demographic questionnaire, the Nurse Work Stress Scale (NWSS), the Psychological Capital Questionnaire-revised (PCQ-R), and the Professional Quality of Life Scale (ProQOL). SPSS 29.0 was used for descriptive statistics and correlation analysis. The PROCESS macro (Model 4) was employed to test the parallel multiple mediation model. HC3 heteroscedasticity-consistent standard errors were used for significance testing of direct effects, and the bias-corrected bootstrap method (5,000 resamples) was applied to estimate the 95% confidence intervals (CI) for indirect effects. Sex, age, marital status, educational level, years of work experience, department, and night shifts per month were included as covariates.

**Results:**

A total of 275 questionnaires were distributed and 274 were collected, yielding 273 valid responses (an effective response rate of 99.3%). The average age of the participating nurses was 32.74 ± 6.94 years. The score for work stress was 83.73 ± 24.15, psychological capital was 84.97 ± 19.53, and compassion fatigue was 58.55 ± 8.07. Pearson correlation analysis showed that work stress was significantly and positively correlated with compassion fatigue (*r* = 0.537, *P* < 0.001), and psychological capital was significantly and negatively correlated with compassion fatigue (*r* = −0.240, *P* < 0.01). The four sub-dimensions of psychological capital (self-efficacy, hope, resilience, and optimism) were also significantly and negatively correlated with compassion fatigue (*r* = −0.137 ~-0.394, *P* < 0.05). After controlling for demographic and work-related covariates, the direct effect of work stress on compassion fatigue remained significant [*c'* = 0.15, *P* < 0.001, *95% CI* (0.10, 0.20)]. Among the four parallel mediators, only resilience showed a significant specific indirect effect [*effect* = 0.058, *95% bootstrap CI* (0.028, 0.091)]. The specific indirect effects through self-efficacy [*effect* = −0.003, *95% CI* (−0.024, 0.009)], hope [*effect* = −0.007, *95% CI* (−0.037, 0.028)], and optimism [*effect* = −0.010, *95% CI* (−0.033, 0.011)] were non-significant.

**Conclusion:**

Among the four psychological capital dimensions, resilience demonstrated the strongest mediating association between work stress and compassion fatigue in this sample. Nursing managers should attach importance to the improvement of the working environment and implement targeted psychological resilience training interventions to reduce the incidence of compassion fatigue and stabilize the nursing workforce.

## 1 Introduction

With the evolving disease spectrum, the aging population, and the ongoing reform of the healthcare system, the public's demand for high-quality and efficient healthcare services continues to rise. The “Healthy China 2030” strategic plan calls for the continuous improvement of healthcare service delivery and quality (1). Clinical nurses, as the largest professional group within the healthcare system and those in closest contact with patients, have a direct impact on patients' care experience and clinical outcomes through the quality of their services (2, 3). The World Health Organization (WHO) noted in its *state of the world's nursing report 2025* (4) that while the global nursing workforce continues to grow, its distribution remains highly uneven, with 100.4 nurses per 10,000 population in high-income countries compared to only 9.3 in low-income countries. By the end of 2025, China had a total of 6.062 million registered nurses, equating to 4.32 registered nurses per 1,000 population (5), indicating a still significant gap from the staffing levels in developed countries.

The relative shortage of nursing staff makes it difficult to meet the public's growing health demands. Clinical nurses face heavy workloads and constantly evolving service requirements, subjecting them to persistently high levels of physical and psychological stress. Research indicates that the global nursing workforce shortage directly leads to increased workloads for nurses, trapping them in a vicious cycle of burnout and attrition (6). Moreover, the greater the number of patients a nurse is responsible for, the more pronounced their occupational stress becomes (7). The high-intensity, high-load nature of nursing work has made this group highly susceptible to psychological problems such as anxiety, depression, andburnout (8).

However, nurses' work stress extends beyond the volume and difficulty of tasks. The extensive interaction with patients and the demand for “caring” in nursing also make it a form of high-intensity emotional labor. As nurses provide professional care and emotional support to patients suffering from illness, their own psychological defenses are continually challenged. This prolonged emotional involvement can easily deplete nurses' psychological resource reserves, leading to a specific form of occupational psychological injury-compassion fatigue (CF).

Compassion fatigue is a mental health problem specific to helping professionals such as nurses. Figley (9) first introduced the concept, defining it as the condition in which healthcare providers, as helpers, are frequently and indirectly exposed to traumatic events and empathize with others, resulting in impaired physical and mental health, diminished interest and capacity for empathy, weakened helping abilities, and severely affected clinician–patient or nurse–patient relationships. Compassion fatigue encompasses two dimensions: job burnout and secondary traumatic stress, manifesting with numerous physiological, behavioral, psychological, and spiritual symptoms. It profoundly impacts nurses' professional identity, self-perception, and wellbeing, leading not only to depression, anxiety, and turnover intention among individual nurses, but also to a reduced capacity for caring, lower patient satisfaction, and an increased risk of adverse events (10, 11). Therefore, effectively preventing the onset of compassion fatigue has become a critical component of safeguarding occupational health in nursing.

Traditional occupational mental health research has largely focused on the pathological mechanisms of psychological problems, emphasizing the elimination of negative emotions. In the early 21st century, scholars such as Martin E.P. Seligman launched the positive psychology movement (12), advocating that psychology should attend to individuals' positive qualities and adopt proactive, preventive approaches to build psychological strengths and resources, rather than waiting for psychological problems to emerge before attempting repair. This marked a shift in perspective within occupational mental health interventions (13). Psychological capital (PsyCap), as an extension of positive psychology into the field of organizational behavior, is defined as an individual's positive psychological state exhibited during growth and development (14). A substantial body of research has demonstrated that psychological capital not only enhances employee performance and job satisfaction but also serves as a crucial psychological buffer against job burnout and work stress (15–17), and exerts a significant protective effect against compassion fatigue (18–20). However, most existing studies have examined psychological capital as a holistic construct in relation to mitigating compassion fatigue (21–23), with little detailed exploration of how each of its four dimensions-self-efficacy, hope, resilience, and optimism-affects compassion fatigue and the magnitude of these effects. Therefore, this study aims to investigate the mediating roles of the four dimensions of psychological capital in the relationship between work stress and compassion fatigue, thereby dissecting the differential effects of each psychological capital dimension on compassion fatigue and providing a basis for targeted interventions.

## 2 Materials and methods

### 2.1 Study design

This study employed a cross-sectional survey design and used convenience sampling to recruit clinical nurses from a tertiary general hospital. The study was approved by biomedical ethical committee of West China Hospital, Sichuan University [Approval No. 2026 (1308)].

### 2.2 Participants

Inclusion criteria: (1) registered nurses holding a valid nursing license; (3) engaged in frontline clinical nursing at the hospital for ≥1 year; (3) informed consent and voluntary participation in the study.

Exclusion criteria: (1) nurses on training; (2) those absent from work during the survey period due to sick, maternity, or other reasons; (3) those with a history of mental illness such as schizophrenia or depression. This exclusion criteria was applied because pre-existing severe mental disorders may independently influence both the perception of work stress and the experience of compassion fatigue, potentially confounding the associations under investigation. The presence of such a history was assessed via a single self-report item embedded in the general demographic questionnaire (“Have you ever been diagnosed by a physician with any of the following mental disorders?”).

### 2.3 Sample size calculation

The required sample size was estimated based on Kendall's principle for estimating sample size in multivariate analysis (24), the sample size should be at least 5–10 times the number of variables. A total of 15 core variables were included in this study (one independent variable, four mediators, and 10 covariates including dummy-coded variables). Based on the minimum requirement of 10 cases per variable, at least 150 cases were needed; accounting for a 20% invalidity rate, the estimated sample size needed 188 cases at least. The final sample of 273 exceeded this minimum requirement.

### 2.4 Research instruments

The general information questionnaire was designed by the researchers based on a review of relevant domestic and international literature. It primarily covered participants' sociodemographic characteristics and work-related information, including gender, age, highest educational level, marital status, professional title, department, years of work experience, and frequency of night shifts.

The Nurse Work Stress Scale (NWSS), developed by Li et al. (25) based on foreign nursing work stress scales and adapted to the Chinese context, comprises 35 items categorized into five types of stressors: issues related to nursing profession and work (7 items), time allocation and workload (5 items), work environment and resources (3 items), patient care (11 items), and management and interpersonal relationships (9 items). Each item is rated on a 4-point Likert scale to assess the impact of different stressors, with higher scores indicating greater perceived stress. In a validation study by Yu et al. (26), the scale demonstrated excellent stability, with Cronbach's alpha coefficients for the five dimensions ranging from 0.80 to 0.89 and test-retest reliability between 0.71 and 0.86. In this study, the Cronbach's alpha coefficient of the scale was 0.971.

The original version of the Psychological Capital Questionnaire was developed by Luthans et al. (27) and comprises 24 items across four dimensions. Luo Hong et al. (28) revised the Chinese version translated by Li et al. (29), removing 4 items to form a revised Psychological Capital Questionnaire(PCQ-R) with 20 items across four dimensions. The four dimensions are self-efficacy (6 items), hope (6 items), resilience (5 items), and optimism (3 items). The questionnaire uses a 6-point Likert scale, with higher scores indicating higher levels of psychological capital. The Cronbach's alpha coefficients for the overall scale and its four dimensions range from 0.707 to 0.927, with test-retest reliability ranging from 0.771 to 0.863, and the scale demonstrates good construct validity. In this study, the Cronbach's alpha coefficient of the scale was 0.964.

The Professional Quality of Life Scale (ProQOL), developed by Stamm (30), consists of three dimensions: compassion satisfaction, job burnout, and secondary traumatic stress, each containing 10 items and rated on a 5-point Likert scale. The job burnout and secondary traumatic stress together increase the risk of compassion fatigue, and the dimersion scores are combined to representing the overall level of compassion fatigue (30, 31). This study used the Chinese version of the ProQOL translated by Chen et al. (32), which has a content validity index of 0.82, an overall Cronbach's alpha coefficient of 0.91, and Cronbach's alpha coefficients for the three dimensions ranging from 0.73 to 0.84. In this study, the Cronbach's alpha coefficient of the scale was 0.770.

### 2.5 Data collection

A standardized electronic questionnaire was generated using the online platform (www.wjx.cn). The researcher contacted the head nurses of each department to explain the purpose and significance of the study and to request permission to approach potential participants. To minimize any perceived pressure to participate arising from this recruitment channel, the following measures were implemented. First, the researcher, rather than the head nurses, directly provided the participants with detailed information and the survey link. Second, explicitly stated that participation was entirely voluntary, that non-participation would have no consequences for employment or performance evaluations, and that participants could withdraw at any time without penalty. Third, all responses were collected anonymously through the online platform, with no collection of IP addresses or other identifying information, and the data were accessible only to the research team.

### 2.6 Statistical analysis

The questionnaire data were exported uniformly from the online platform in Excel format. After excluding invalid questionnaires and verifying the data, they were imported into IBM SPSS Statistics 29.0 for data processing and analysis. Categorical variables, such as clinical nurses' demographic and work-related characteristics, were described using frequencies and percentages (%). For continuous variables, including work stress, psychological capital, and compassion fatigue, skewness and kurtosis were examined to assess conformity to normal distribution; data meeting or approximating normal distribution were described using mean ± standard deviation (x̄ ± s). Harman's single-factor test was conducted by performing an unrotated principal component analysis on all measurement items, with the variance explained by the first principal component below 40% serving as the criterion for the absence of serious common method bias (33). Pearson correlation coefficients were used to analyze the pairwise correlations between total work stress scores, the four dimension scores of psychological capital, and total compassion fatigue scores. Furthermore, a parallel multiple mediator model was tested using the PROCESS macro (Version 5.0, Model 4) module (34). In this model, work stress served as the independent variable (X), compassion fatigue as the dependent variable (Y), and the four dimensions of psychological capital—self-efficacy, hope, resilience, and optimism—as parallel mediators (M1–4). Based on prior literature, the following demographic and work-related characteristics were entered as covariates to control for their potential confounding effects: sex, age, marital status, educational level, years of work experience, department, and night shifts per month. Unordered multi-categorical covariates (marital status, department) were dummy-coded prior to entry. The Breusch–Pagan test indicated the presence of heteroskedasticity (robust test, *P* = 0.024); therefore, HC3 heteroscedasticity-consistent standard errors were employed for significance testing of regression coefficients. The indirect effects were estimated using bias-corrected bootstrap 95% confidence intervals (*CI*) based on 5,000 resamples. An indirect effect was considered statistically significant if the 95% *CI* did not include zero.

## 3 Results

A total of 275 nurses were invited to participate in this study. Of the 274 questionnaires returned, one was excluded due to patterned responses (selecting the same option throughout), yielding 273 valid questionnaires, with an effective response rate of 99.3%.

### 3.1 Participants' characteristics

Among the 273 clinical nurses surveyed, the majority were female (89.38%), with a mean age of 32.74 ± 6.94 years. Married nurses accounted for 57.88%, 67.77% held a bachelor's degree or higher, and 45.05% had 10 years or more of frontline nursing experience. The sample covered a wide range of departments, with nurses from high-risk and high-intensity settings such as emergency, ICU, and operating room accounting for 24.91%. Additionally, approximately 76.19% of the nurses were required to work night shifts at varying frequencies during the survey period, with 34.43% had five or more night shifts per month. See Table 1 for details.

**Table 1:** Distribution of general demographic and work-related characteristics of clinical nurses (*N* = 273).

Variables	Groups	*n*	%
Sex	Male	29	10.62
	Female	244	89.38
Age	≤ 25	33	12.09
	25~30	84	30.77
	30~35	62	22.71
	>36	94	34.43
Marital status	Unmarried	106	38.83
	Married	158	57.88
	Divorced or widowed	9	3.29
Educational level	Associate degree or below	88	32.23
	Bachelor's degree	171	62.64
	Master's degree or above	14	5.13
Years of work experience	≤ 3	51	18.68
	3~10	99	36.26
	>10	123	45.05
Professional title	None	53	19.41
	Junior title	100	36.63
	Intermediate title	99	36.26
	Senior title	21	7.69
Department	Internal medicine	92	33.70
	Surgery	81	29.67
	Emergency/ICU/operation room	68	24.91
	Others	32	11.72
Night shifts per month	0	65	23.81
	1~4	114	41.76
	4~8	76	27.84
	>8	18	6.59

ICU, intensive care unit.

### 3.2 Scale scores and correlation analysis

The skewness and kurtosis of the total scores for work stress, psychological capital, compassion fatigue, and the four dimensions of psychological capital were calculated to assess normality. Based on the data normality criteria proposed by Hair et al. (35) data can be considered approximately normally distributed when the absolute values of both skewness and kurtosis are less than 2. Accordingly, scale scores are presented as mean ± standard deviation. Harman's single-factor test showed that the first unrotated factor explained 29.27% of the total variance, which is below the 40% threshold, indicating that serious common method bias was not present in this study. Among the respondents, the clinical nurses' work stress score was 83.73 ± 24.15, psychological capital score was 84.97 ± 19.53, and compassion fatigue score was 58.55 ± 8.07. See Table 2 for details.

**Table 2:** Scale scores and normality test results.

Scale/dimentions	Mean	SD	Skew	Kurtosis
NWSS	83.73	24.15	−0.05	−0.58
PCQ-R	84.97	19.53	−0.23	−0.15
Self-efficacy	25.83	7.32	−0.48	−0.09
Hope	25.62	6.88	−0.27	−0.36
Resilience	20.28	4.18	0.50	0.70
Optimism	13.25	3.34	−0.33	−0.09
Compassion Fatigue(calculated by ProQOL)	58.55	8.07	−0.15	0.57

Pearson correlation analysis was conducted to examine the relationships among work stress, psychological capital (total score and sub-dimensions), and compassion fatigue. The results showed that work stress was positively correlated with compassion fatigue, while psychological capital and its sub-dimensions were all negatively correlated with compassion fatigue. Work stress was also negatively correlated with psychological capital and its sub-dimensions. Notably, the correlation of resilience—a sub-dimension of psychological capital—with work stress and compassion fatigue was stronger than that of the total psychological capital score. The PCQ-R total score was strongly correlated with each of its four subdimensions, and the intercorrelations among the four subdimensions were all moderate to strong. The results are presented in Table 3.

**Table 3:** . Pearson correlation analysis among work stress, psychological capital, and compassion fatigue.

Scale/ dimentions	NWSS	PCQ-R	Self-efficacy	Hope	Resilience	Optimism	Compassion fatigue
NWSS	—						
**PCQ-R**	−0.377^***^	—					
Self-efficacy	−0.259^***^	0.917^***^	—				
Hope	−0.357^***^	0.951^***^	0.837^***^	—			
Resilience	−0.475^***^	0.795^***^	0.578^***^	0.681^***^	—		
Optimism	−0.305^***^	0.885^***^	0.722^***^	0.816^***^	0.730^***^	—	
**Compassion fatigue**	0.537^***^	−0.240^**^	−0.128^*^	−0.195^**^	−0.419^***^	−0.198^**^	—

^*^indicates *P* < 0.05; ^**^indicates *P* < 0.01; ^***^indicates *P* < 0.001.

### 3.3 Parallel multiple mediation analysis

The parallel multiple mediator model, controlling for sex, age, marital status, educational level, years of work experience, department, and night shifts per month, explained 35.18% of the variance in compassion fatigue (*R*^2^ = 0.604, adjusted *R*^2^ = 0.364, *F* = 9.934, *P* < 0.001). Prior to the analysis, multicollinearity diagnostics were conducted for all predictors. Variance inflation factors (VIFs) ranged from 1.0815 to 5.37, with all values below 10. With the exception of the hope dimension, which had a VIF slightly above 5 (*VIF* = 5.37), all other VIFs were below 5, indicating that overall multicollinearity was within an acceptable range.

The direct effect of work stress on compassion fatigue was significant [ć = 0.15, *se* = 0.025, *P* < 0.001, *95% CI* (0.10, 0.20)]. The total indirect effect of work stress on compassion fatigue through the four psychological capital dimensions was also significant [*effect* = 0.038, *Bootstrap SE* = 0.011, *95% CI* (0.017, 0.061)].

Regarding the specific indirect effects, only resilience emerged as a significant mediator. The specific indirect effect through resilience was 0.058 [*Bootstrap SE* = 0.016, *95% CI* (0.028, 0.091)]. In contrast, the specific indirect effects through self-efficacy [*effect* = −0.003, *95% CI* (−0.024, 0.009)], hope [*effect* = −0.007, *95% CI* (−0.037, 0.028)], and optimism [*effect* = −0.010, *95% CI* (−0.033, 0.011)] were all non-significant, as their 95% bootstrap confidence intervals included zero.

As shown in Table 4, among the four psychological capital dimensions, only resilience was a significant negative predictor of compassion fatigue when controlling for work stress and the other mediators [*coeff* = −0.71, *P* < 0.001, *95% CI* (−1.039, −0.365)]. The direct paths from self-efficacy, hope, and optimism to compassion fatigue were all non-significant (*P* > 0.05). None of the demographic covariates had significant unique effects on compassion fatigue (*P* > 0.05).

**Table 4:** Direct effects, indirect effects, and bootstrap confidence intervals for the parallel multiple mediation model.

Effect type	Path/mediator	Coeff/effect	SE/boot SE	95% CI	P
Direct effect (c')	Work stress → Compassion fatigue	**0.15**	**0.025**	**[0.10, 0.20]**	**< 0.001**
Total indirect effect	X → All Ms → Y	**0.038**	**0.011**	**[0.017, 0.061]**	—
Specific indirect effects					
	Work stress → Self-efficacy → Compassion fatigue	−0.003	0.008	[−0.024, 0.009]	—
	Work stress → Hope → Compassion fatigue	−0.007	0.016	[−0.037, 0.028]	—
	Work stress → Resilience → Compassion fatigue	**0.058**	**0.016**	**[0.028, 0.091]**	—
	Work stress → Optimism → Compassion fatigue	−0.010	0.011	[−0.033, 0.011]	—
Paths from mediators to Y					
	Self-efficacy → Compassion fatigue	0.042	0.122	[−0.198, 0.282]	0.729
	Hope → Compassion fatigue	0.073	0.187	[−0.299, 0.442]	0.696
	Resilience → Compassion fatigue	–**0.707**	**0.181**	**[**–**1.063**, –**0.35]**	**< 0.001**
	Optimism → Compassion fatigue	0.256	0.281	[−0.297, 0.809]	0.363

*CI*, confidence interval. Covariates: sex, age, marital status, educational level, years of work experience, department, and night shifts per month. Indirect effects were evaluated using bias-corrected bootstrap 95% confidence intervals. Bolded effects are statistically significant (*CI* does not include zero for indirect effects; *P* < 0.05 for direct paths).

## 4 Discussion

### 4.1 Compassion fatigue among clinical nurses

The results of this study showed that the surveyed clinical nurses reported considerable levels of work stress and compassion fatigue, consistent with the pattern observed in related domestic and international studies (31, 36). Specifically, the overall work stress score of the clinical nurses in this study was lower than the 102.83 ± 15.93 reported in a previous survey of emergency department nurses (37), while the psychological capital score was slightly higher than that of emergency nurses (81.46 ± 12.17). The compassion fatigue score was also lower than levels typically reported in high-acuity settings (31). These observed differences are consistent with the possibility that nurses working in high-acuity departments such as the emergency department may be exposed to greater work stress, which could contribute to lower psychological capital and higher compassion fatigue relative to the broader clinical nursing population.

From an occupational health and safety perspective, work stress in nursing should be recognized as a psychosocial occupational risk factor that arises from the work environment rather than from individual deficits. Clinical nurses endure not only the physiological burden of rotating night shifts and irregular daily routines, but also face psychological and emotional demands arising from patients' changing conditions, death-related events, nurse-patient communication, and emotional labor. Sinclair et al. (38) noted that frequent exposure to death, tense clinician–patient relationships, and the relative inadequacy of medical resources constitute major sources of chronic psychosocial hazard for the nursing population. These hazards, when left unmitigated, can produce measurable occupational health outcomes, of which compassion fatigue-operationalized in this study as the combined toll of burnout and secondary traumatic stress-is a critical example. Framing compassion fatigue as an occupational health outcome, rather than merely as an individual psychological reaction, underscores the responsibility of healthcare organizations to systematically monitor, prevent, and mitigate this condition as part of their occupational health and safety management systems.

### 4.2 The role of psychological capital in the pathway from work stress to health outcome

The *Pearson* correlation analysis showed that work stress, psychological capital, and its four dimensions (self-efficacy, hope, resilience, and optimism) were all significantly correlated with compassion fatigue scores. Previous studies have confirmed that work stress is an important risk factor for compassion fatigue among clinical nurses. A large-sample cross-sectional survey of emergency department nurses in China (*N* = 1,014) found that occupational stress was one of the independent predictors of compassion fatigue (39). Similar findings have been reported in studies from other departments: some researchers noted that nurses experiencing occupational stress had a higher proportion of compassion fatigue (*OR* 2.668, *95% CI* 0.799–8.898) and significantly higher levels of job burnout and secondary traumatic stress (40). A study of 13,936 Chinese nurses conducted during the COVID-19 pandemic further revealed the impact of work stress on various dimensions of professional quality of life. The structural equation model demonstrated that work stress significantly and positively predicted job burnout (β = 0.334, *P* < 0.001) and secondary traumatic stress (β = 0.222, *P* < 0.001), with secondary traumatic stress being a key component of compassion fatigue (41).

Psychological capital, a positive psychological resource comprising hope, self-efficacy, resilience, and optimism, is an important protective factor that buffers against compassion fatigue (42). Relevant studies have demonstrated that the higher the level of psychological capital, the less susceptible nurses are to compassion fatigue and burnout. One study found that positive psychological capital not only had a significant negative effect on compassion fatigue (β = −0.29, *P* < 0.001) but also further influenced burnout through the partial mediating effects of compassion fatigue and compassion satisfaction (43). Research on Chinese clinical nurses has also indicated that psychological capital is an important correlate of compassion fatigue (36). However, the roles played by different dimensions of psychological capital are not identical. One study showed that the low resilience dimension of psychological capital increased the risk of compassion fatigue by 2.14 times (*OR* = 2.14, *P* = 0.007) (44). Another study of nurses in a Turkish general hospital found that self-efficacy and optimism explained 26% of the variance in the job burnout regression model, which increased to 45% after adding compassion satisfaction; however, in the compassion fatigue model, self-efficacy accounted for only 5% of the variance, and the mediating effect of compassion satisfaction was not significant (45). This suggests that the pathways through which psychological capital affects different occupational health outcomes may differ.

Multiple studies have demonstrated a stable negative association between work stress and psychological capital. That is, the higher the work stress perceived by nurses, the lower their level of psychological capital tends to be, and this relationship is relatively consistent across different departments and contexts. A study of 288 ICU nurses found that psychological capital was significantly and negatively correlated with work stress (46). Another study of pediatric ward nurses also showed a significant negative relationship between psychological capital and occupational stress (β = −0.23, *P* < 0.001) (47). Further research suggests that work stress is not merely an external correlate of psychological capital; it may also alter psychological capital itself through the ongoing depletion of individual resources. Working conditions characterized by high effort, high overcommitment, and low reward can also weaken nurses' psychological capital; conversely, increased rewards are associated with enhanced psychological capital (48). This bidirectional relationship has critical implications for occupational risk management: it indicates that the work environment does not simply interact with an individual's stable psychological traits, but actively shapes and depletes those psychological resources over time. Consequently, the prevention of compassion fatigue cannot rely solely on strengthening individual coping capacities; it must address the modifiable workplace stressors at their source.

### 4.3 Resilience as a key mediator between work stress and compassion fatigue

By constructing a parallel multiple mediation model, this study examined the differential roles of the four sub-dimensions of psychological capital in the pathway from work stress to compassion fatigue. The results indicated that, among the four dimensions, only resilience emerged as a statistically significant mediator in this relationship, whereas the indirect effects through self-efficacy, hope, and optimism did not reach statistical significance in the present sample.

From the perspective of Conservation of Resources theory, stress occurs when individuals face resource loss, actual resource loss, or fail to obtain adequate returns after investing resources (49). In clinical settings, nurses frequently encounter highly emotionally demanding events such as patient suffering, deterioration, and death. In these situations, what is most immediately required is not positive expectations about future outcomes or general confidence, but rather the capacity to maintain basic psychological functioning after experiencing emotional shock and to restore adaptive equilibrium as rapidly as possible. The core definition of resilience is precisely the ability to “bounce back from adversity,” which conceptually aligns closely with the psychological mechanism that blocks the transformation of stress into compassion fatigue.

Mealer et al. (50) found in a study of intensive care unit (ICU) nurses that those with high resilience had a significantly lower incidence of post-traumatic stress disorder and were able to restore psychological equilibrium more quickly after negative events such as patient death. Similar differential effects among sub-dimensions have been supported by research in other fields. A study of South African social and commercial entrepreneurs found that only hope and resilience mediated the relationship between entrepreneurial intention and entrepreneurial action, whereas the mediating effects of self-efficacy and optimism were not significant (51). Thus, resilience may constitute a more fundamental psychological resource in nurses' coping with compassion fatigue, capable of slowing the loss of emotional resources under persistent stress and reducing the risk of work stress transforming into compassion fatigue.

Critically, however, this finding should not be interpreted as placing the burden of prevention on individual nurses to “become more resilient.” Rather, it points to the responsibility of healthcare organizations to systematically protect, maintain, and restore the resilience of their nursing workforce through workplace-level strategies. These strategies can be organized within an occupational risk management framework comprising three tiers: primary prevention focuses on controlling psychosocial hazards at their source, including adequate nurse staffing, reasonable workload allocation, shift schedules that minimize circadian disruption, and protected rest periods; secondary prevention involves organizationally supported resilience-building programs, such as mindfulness-based stress reduction (MBSR) (52) and structured post-trauma debriefing (e.g., Balint Groups) (53), integrated into routine occupational health services; tertiary prevention entails regular, confidential psychological health surveillance and accessible, stigma-free occupational mental health services, supported by a culture of psychological safety. These multi-tiered strategies collectively shift the focus from individual coping to systemic occupational health protection.

### 4.4 Implications for occupational mental health policy and nursing management

The findings of this study carry several implications for occupational health policy and nursing management. First, healthcare organizations should integrate psychosocial risk assessment—including the monitoring of work stress, compassion fatigue, and psychological capital—into routine occupational health audits, parallel to existing physical health and safety assessments. Second, hospital administrators and nursing managers should develop organizational support systems that explicitly address psychological health alongside physical safety. This includes establishing reasonable nurse staffing levels, optimizing workload distribution, improving night-shift compensation and scheduling, and ensuring that nurses have access to protected rest periods during demanding shifts. Third, human resource policies should recognize compassion fatigue as a legitimate occupational health concern that warrants institutional response, including the provision of accessible occupational mental health services. At the broader policy level, this study supports the inclusion of psychosocial risk management and mental health protection standards in healthcare accreditation and regulatory frameworks.

### 4.5 Study limitations and future directions

This study has the following limitations that suggest directions for future research. (1) A cross-sectional design was employed. Although causal pathways were constructed through theoretical deduction, causality between variables cannot be rigorously established in the temporal dimension. Future research could adopt longitudinal designs to further verify the dynamic protective effects of resilience. (2) All variables were measured using self-report questionnaires, which may inevitably be subject to recall bias. Although Harman's single-factor test was conducted and suggested that common method bias was not a serious threat in this dataset, this test carries recognized psychometric limitations and provides only a diagnostic indication rather than a definitive correction. Future studies could adopt more robust procedural and statistical remedies, such as incorporating structural marker variables, to better assess and control for common method variance. Additionally, incorporating objective, multi-source data such as peer evaluations, turnover statistics, and physiological indicators could further enhance the internal validity of the research. (3) Due to time and resource constraints, convenience sampling was used to recruit nurses from a single tertiary hospital. This single-center design limits the wider generalizability of the findings, as the sample may not adequately represent nurses working in other hospital tiers, geographic regions, or healthcare systems where work stressors and coping resources may differ substantially. Multi-center, cross-regional studies are needed to validate and extend these findings. (4) Although baseline demographic and work-related characteristics were controlled as covariates, other unmeasured confounders may still influence the relationships under investigation. (5) Future research employing multilevel modeling could simultaneously examine individual-level and organization-level predictors, providing a more comprehensive understanding of how workplace factors contribute to occupational health outcomes in nursing.

## 5 Conclusion

This study found that work stress, as a psychosocial occupational risk factor, exerts both direct and indirect effects on compassion fatigue. Among the dimensions of psychological capital, resilience emerged as the sole significant mediator in this pathway. These findings suggest that the prevention of compassion fatigue should prioritize systemic workplace-level strategies to control psychosocial hazards at their source, including adequate nurse staffing, reasonable workload allocation, ergonomic shift scheduling, and organizational support. Targeted resilience-building interventions may serve as a complementary approach to strengthen nurses' capacity to manage unavoidable occupational stressors. A dual strategy that combines primary prevention of workplace stressors with evidence-based resilience support may more effectively protect the occupational health and well-being of the nursing workforce.

## Data Availability

The data that support the findings of this study are available from the corresponding author upon reasonable request.
